# Assessment of ground transportation stress in juvenile Kemp’s ridley sea turtles (*Lepidochelys kempii*)

**DOI:** 10.1093/conphys/cov071

**Published:** 2016-03-01

**Authors:** Kathleen E. Hunt, Charles J. Innis, Adam E. Kennedy, Kerry L. McNally, Deborah G. Davis, Elizabeth A. Burgess, Constance Merigo

**Affiliations:** 1 John H. Prescott Marine Laboratory, Research Department, New England Aquarium, Boston, MA 02110, USA; 2 Animal Health Department, New England Aquarium, Boston, MA 02110, USA; 3 Rescue and Rehabilitation Department, New England Aquarium, Boston, MA 02110, USA; 4 Idexx Laboratories, 3 Centennial Drive, North Grafton, MA 01536, USA

**Keywords:** Corticosterone, glucose, Kemp’s ridley sea turtle, sea turtles, stress physiology, transportation stress

## Abstract

Sea turtle rehabilitation centres frequently transport sea turtles for long distances to move animals between centres or to release them at beaches, yet there is little information on the possible effects of transportation-related stress (‘transport stress’) on sea turtles. To assess whether transport stress is a clinically relevant concern for endangered Kemp’s ridley sea turtles (*Lepidochelys kempii*), we obtained pre-transport and post-transport plasma samples from 26 juvenile Kemp’s ridley sea turtles that were transported for 13 h (*n* = 15 turtles) or 26 h (*n* = 11 turtles) by truck for release at beaches. To control for effects of handling, food restriction and time of day, the same turtles were also studied on ‘control days’ 2 weeks prior to transport, i.e. with two samples taken to mimic pre-transport and post-transport timing, but without transportation. Blood samples were analysed for nine clinical health measures (pH, pCO_2_, pO_2_, HCO_3_, sodium, potassium, ionized calcium, lactate and haematocrit) and four ‘stress-associated’ parameters (corticosterone, glucose, white blood cell count and heterophil-to-lymphocyte ratio). Vital signs (heart rate, respiratory rate and cloacal temperature) were also monitored. Corticosterone and glucose showed pronounced elevations due specifically to transportation; for corticosterone, this elevation was significant only for the longer transport duration, whereas glucose increased significantly after both transport durations. However, clinical health measures and vital signs showed minimal or no changes in response to any sampling event (with or without transport), and all turtles appeared to be in good clinical health after both transport durations. Thus, transportation elicits a mild, but detectable, adrenal stress response that is more pronounced during longer durations of transport; nonetheless, Kemp’s ridley sea turtles can tolerate ground transportation of up to 26 h in good health. These results are likely to depend on specific transportation and handling protocols.

## Abstract (precis)

Juvenile Kemp’s ridley sea turtles were studied before and after ground transportation to assess potential transportation stress. Turtles appeared mildly stressed after a 13h transport (mild elevations in glucose) and moderately stressed by a 26h transport (more pronounced elevations in corticosterone and glucose), but remained in good clinical health.

## Introduction

Sea turtle rehabilitation centres must often transport sea turtles over long distances by vehicle or aircraft, e.g. from stranding sites to rehabilitation centres, to other centres for further treatment and rehabilitation or (later) to release rehabilitated animals at beaches. Given that sea turtles breathe air, it is common management practice to transport sea turtles out of water in small crates or boxes. In North America, hundreds of sea turtles have been transported in recent years after mass stranding and rescue events (e.g. cold-stunning events, Deepwater Horizon oil spill), and such transportation events (‘transports’) can sometimes exceed 24 h. For example, juvenile Kemp’s ridley sea turtles (*Lepidochelys kempii*), a critically endangered species (IUCN, 2010), have been stranding in increasing numbers in the months of October–December on Cape Cod, MA, USA (Allman, 1998; Gerle *et al.*, 2000; Still *et al.*, 2005), and many of these turtles are later transported as far as Georgia or Florida for rehabilitation and/or release into waters with appropriate temperatures. However, there is little information available on potential effects of transportation-related stress (‘transport stress’) on sea turtle health or physiology. Transport stress is particularly a concern for critically ill turtles and for those transports that occur the day of or the day before release, because ideally turtles should be in optimal health at the moment of release. If transportation on the day of release results in excessive physiological stress, the possibility exists that turtles may be released in a state of suboptimal health.

Transportation is a known stressor for virtually all vertebrates (Grandin, 2007; Shim *et al.*, 2009; Vasquez-Galindo *et al.*, 2013), including terrestrial turtles (Fazio *et al.*, 2014). If transport stress is extreme there can be immediate impacts on clinical health (e.g*.* via dehydration and exhaustion), which are typically detectable via changes in standard clinical health parameters, such as heart rate, respiratory rate, plasma electrolytes, haematocrit, blood gases, plasma pH, lactate and other blood chemistry measures (Knowles and Warriss, 2007). Transport stress can also have more subtle effects via stimulation of an adrenal stress response (Knowles and Warriss, 2007). In most wildlife species, transportation by motorized methods (e.g*.* truck, aircraft) results in a sharp increase in adrenal hormones, including the catecholamines and corticosterone (and/or cortisol, depending on the species; Teixeira *et al.*, 2007; Dickens *et al.*, 2010). Corticosterone, the best studied of these hormones, reorients available energy toward emergency physiological responses and has downstream effects that include increased blood glucose, changes in white blood cell count (WBC), elevations in heterophil-to-lymphocyte ratio (H/L ratio) and a host of other physiological changes (Widmaier, 1990; Mizock, 1995; Bentley, 1998; Sapolsky *et al.*, 2000; Wingfield and Romero, 2011). Generally, a brief elevation in corticosterone (minutes to hours) is not necessarily detrimental, serving to redirect available energy toward an ‘emergency’ life-history state (e.g*.* increased foraging and locomotion, decreased reproduction and growth). However, in virtually all vertebrates studied, a prolonged elevation in corticosterone can later result in negative health effects, such as increased vulnerability to disease, inhibition of growth and reproduction, immunosuppression, alterations in memory and changes in behaviour (Bentley, 1998; Sapolsky *et al.*, 2000; Wingfield and Romero, 2011).

From a clinical perspective, some degree of physiological stress is inevitable when handling and transporting wildlife (Dickens *et al.*, 2010; Parker *et al.*, 2011). However, transport stress should be minimized where possible, via assessment of the magnitude and degree of transport stress with different durations and with specific transport protocols (handling method, transport environment etc.). Ultimately, assessment of transport stress can be approached via two avenues: (i) immediate health impacts of a transport event, via assessment of post-transport health status (blood chemistry, blood gases, haematocrit and vital signs); and (ii) potential long-term impacts of a physiological stress response, via assessment of corticosterone and/or its immediate downstream targets (blood glucose, WBC, H/L ratio etc.; Mizock, 1995; Knowles and Warriss, 2007; Mormède *et al.*, 2007; Davis *et al.*, 2008; Martin, 2009).

Our goal in the present study was to determine whether half-day (∼12 h) or full-day (∼24 h) transport causes adverse physiological effects in Kemp’s ridley sea turtles. Turtles were studied before and after transport, and comparisons were made with control events when turtles were handled but not transported. We focused on the following parameters: (i) state of health before vs. after transport (assessed via electrolytes, blood gases, plasma pH, lactate, haematocrit and vital signs); and (ii) evidence of a detectable physiological stress response, as indicated by changes in corticosterone, glucose, WBC or H/L ratio before vs. after transport.

## Materials and methods

### Study animals

Experimental design involved opportunistic study of juvenile Kemp’s ridley sea turtles (*L. kempii*) that were being transported from the New England Aquarium’s sea turtle rehabilitation centre (NEAq; Quincy, MA, USA) to the coasts of Virginia or Florida for release. All turtles had stranded in a cold-stunned state on the northern shore of Cape Cod during the previous autumn (admission dates of 31 October to 17 December) and were housed in rehabilitation tanks for 4–7 months before the transport events described in the present study. In this species, behaviour and corticosterone levels of cold-stunned juveniles typically returns to presumed normal levels after ∼1 month in captivity (Hunt *et al.*, 2012). All turtles were estimated to be 2–3 years of age based on carapace length at the time of stranding, in comparison to known-age conspecifics (B. Higgins, National Marine Fisheries Service, personal communication; Avens and Snover, 2013). While in captivity, turtles were maintained in saltwater tanks at ∼26°C, the average of the preferred temperature range for this species (Renaud, 1995), and were monitored daily, with food offered twice daily (herring and squid). Prior to the present study, all turtles were judged by veterinary staff to be stable clinically and in good condition based on serial physical examinations, haematological and plasma biochemical analysis and radiographic evaluation.

The NEAq sea turtle rehabilitation programme and associated research were conducted with the authorization of the US Department of the Interior Fish and Wildlife Service (permit number TE-697823), in compliance with guidelines of the NEAq Animal Care and Use Committee (IACUC protocol 2012-03). Turtle transports and beach releases were performed in compliance with all applicable local, state and federal regulations and guidelines.

### Study design and transportation protocols

In order to control for potential effects of handling, food deprivation and time of day, each turtle was studied twice, first on a ‘control day’, during which the turtle was handled twice (and not fed) but without being transported, followed 2 weeks later by a ‘transport day’, during which the turtle was handled before and after transport to a beach for release. On each day, a pre-event and a post-event sample was taken. Each turtle thus had four samples taken, an initial pair termed the ‘pre-control’ and ‘post-control’ samples taken on the control day, and later, a second pair of samples termed the ‘pre-transport’ and ‘post-transport’ samples taken on the transport day. In 2012, 11 turtles were studied on both a 13 h control day and 13 h transport day; these turtles are referred to as the ‘13 h turtles’. In 2013, 15 turtles were studied on a 23 h control day and 26 h transport day (the minor difference in control vs. transport duration was because of traffic); these turtles are referred to as the ‘26 h turtles’. Note that the inclusion of a ∼24 h study duration enabled us to control for the effect of time of day, because any changes seen in samples taken roughly 24 h apart are not likely to be due to time of day. The 13 h turtles were slightly larger than the 26 h turtles [13 h turtles, 5.35 ± 0.31 kg body mass (mean ± SEM), range 3.35–8.00 kg; 26 h turtles, 3.40 ± 0.31 kg body mass (mean ± SEM), range 2.35–5.15 kg]. There were no apparent differences between 13 and 26 h turtles in clinical health, behaviour or any other parameters.

#### First sampling (pre-control and pre-transport samples)

Turtles were removed from their tanks by placing a net in the water, gently scooping up the turtle, removing it from the net by hand, and holding the turtle slightly tipped for ∼5 s to allow water to drain from the mouth. Turtles were then carried to an examination room, where a 2–3 ml heparinized blood sample was immediately collected from the external jugular vein as previously described (Innis *et al.*, 2007, 2009), followed by measurements of cloacal temperature, heart rate and respiratory rate. Most turtles were also weighed to the nearest 0.1 kg on a digital scale and measured for straight carapace length (notch to tip) and straight carapace width (at widest point). Any turtles that had been weighed and measured within the previous 7 days were not weighed and measured again. Once handling was completed, turtles were either returned to their tanks immediately (control days) or were placed in individual crates and loaded into a truck (transport days). Turtles were not fed on either control days or transport days.

To minimize effects of acute handling stress on corticosterone levels (Moore *et al.*, 1991; Dunlap and Wingfield, 1995; Romero and Reed, 2005), all blood sampling and handling was timed from ‘time zero’, i.e. time of first disturbance, defined as the time the net was first placed into that turtle’s tank or (for post-transport samples only) the time the turtle’s crate was opened. ‘Bleeding time’, i.e. elapsed time from time zero to the time the blood sample was taken, averaged 2.09 ± 0.12 min (combining all turtles and all events). Handling time, defined as the elapsed time from time zero to the time turtle was released back to tank or placed back in crate, averaged 9.00 ± 0.40 min on control days. Handling time was not always tracked on transport days because of the logistics of loading turtles into trucks and releasing them at beaches, but was very similar to that of control days.

#### Transportation protocol

On transport days, once blood sampling and veterinary examinations were concluded, turtles were placed in individual hinged-top plastic crates with padding at the bottom (foam pad and/or towels). All crates had air holes in the sides for ventilation. Once in the crates, turtles received subcutaneous fluid therapy (lactated Ringer’s solution, 10 ml/kg), and shells and flippers were coated with a thin layer of water-soluble lubricant to help prevent dehydration of the skin. Crates were then closed and loaded onto the transportation vehicles, which in all cases were large-size passenger sport utility vehicles with a climate-controlled environment, i.e*.* noise, temperature and vibration were similar to those of typical passenger vehicles. Vehicle ambient temperature was set to 26°C. Trucks were then driven either 13 h from Quincy, MA to Vauclose Shores beach in Machipongo, VA or 26 h from Quincy, MA to Little Talbot Island State Park, Jacksonville, FL, USA.

#### Second sampling (post-control and post-transport samples)

On control days, turtles were again removed from their tanks after the prescribed time interval and examined and sampled again as described above [see ‘*First sampling (pre-control and pre-transport samples)*’]. On transport days, the post-transport sampling occurred as soon as possible upon arrival at the release site. Turtle crates were lined up outside the truck under shade, and a portable handling station and laboratory (for processing blood samples) was set up and post-transport samples taken as described above. Turtles were released to the sea promptly after the post-transport sampling was complete. All turtles appeared active and alert upon release, crawled actively when placed on beach sand and swam well when they reached the surf.

#### Event dates and timing

For the 13 h turtles, pre-control samples were collected on 23 May 2012 between 06.15 and 09.16 h, post-control samples later that day between 19.04 and 21.10 h, pre-transport samples on 20 June 2012 between 04.25 and 06.23 h, and post-transport samples later that day from 17.08 to 19.29 h. For the 26 h turtles, pre-control samples were collected between 09.31 and 09.59 h on 20 March 2013, post-control samples between 08.39 and 09.04 h on 21 March 2013, pre-transport samples between 08.45 and 09.20 h on 6 April 2013, and post-transport samples between 10.41 and 11.14 h on 7 April 2013. Total event duration, defined as the elapsed time from collection of the first blood sample to collection of the second blood sample from the same turtle, averaged 12.33 ± 0.08 h for the 13 h control day, 12.90 ± 0.04 h for the 13 h transport day, 23.07 ± 0.02 h for the 23 h control day and 25.89 ± 0.01 h for the 26 h transport day (means ± SEM).

### Blood analyses: i-STATs, complete blood counts and corticosterone assays

Whole-blood samples were immediately divided into three portions, as follows: (i) 0.20 ml was used for point-of-care clinical chemistry analyses of electrolytes, glucose, lactate, blood gases and plasma pH, using an i-STAT analyser as described in the next subsection; (ii) approximately 0.5–1.0 ml of whole blood was shipped to an outside laboratory (IDEXX Reference Laboratories, North Grafton, MA, USA) for a complete blood count; and (iii) the remaining blood was refrigerated within 10 min and later (within 3 h) centrifuged at 1500***g*** for 5 min, with plasma pipetted to a cryovial and frozen for later corticosterone analysis at our laboratory in Boston, MA, USA (see ‘*Corticosterone assay*’ subsection below for detailed methods). For post-transport samples taken at remote locations, whole blood was shipped chilled (not frozen) on ice packs and plasma was shipped frozen on dry ice to the appropriate laboratories.

#### Clinical chemistry (i-STAT analyses)

Immediately upon collection of the blood sample, whole blood was loaded directly into both an i-STAT CG4+ Test Cartridge and to an i-STAT CG8+ Test Cartridge, which were then analysed using a portable battery-powered hand-held point-of-care analyser, VetScan i-STAT^®^1 Analyzer Model 300A (Abbott Point Of Care, Princeton, NJ, USA). Two i-STAT machines were used in the study; each turtle’s control and transport samples were always analysed by the same i-STAT machine, and generally, data appeared comparable between machines (data not shown). The CG4+ cartridges measured plasma pH, partial pressure of oxygen (pO_2_), partial pressure of carbon dioxide (pCO_2_), HCO_3_ (bicarbonate) and lactate. The CG8+ cartridges measured sodium, potassium, ionized calcium (iCa) and glucose, and also produced back-up data on pH, pO_2_, pCO_2_ and HCO_3_. Data for pH, pO_2_, pCO_2_ and HCO_3_ were taken from the first cartridge analysed, which in almost all cases was the CG4+ cartridge. In two instances (one 13 h turtle and one 26 h turtle), the CG4+ cartridge initially failed; hence, the CG8+ cartridge was then analysed first while a new CG4+ cartridge was prepared, in order to minimize delays in obtaining blood gas measurements. All pH, pO_2_ and pCO_2_ data were temperature corrected (TC) for the cloacal temperature of each turtle, and iCa data were pH corrected using previously described formulas (Keller *et al.*, 2012). Bicarbonate concentration in blood samples was calculated by use of the Henderson–Hasselbalch equation, pH_TC_ and pCO_2TC_, with αCO_2_ and p*K* values calculated by previously described species-specific equations for Kemp’s ridley sea turtles (Stabenau and Heming, 1993). As it is presently unknown how rapidly samples must be analysed so as to obtain accurate blood gas measurements for sea turtles, the time lag between blood sample collection and loading of the cartridges to the i-STAT machine was recorded individually for each turtle and reported here as ‘CG4 lag time’ and ‘CG8 lag time’ for the two cartridges separately. i-STAT machines process cartridges in a sequence of automated steps that include cartridge identification, calibration and analysis; for logistical reasons, the time point chosen for consistent measurement of lag time was the moment when the calibration step began.

#### Complete blood counts

Complete blood counts, including haematocrit, total WBC and white blood cell differential count, were determined within 18 h for locally collected samples and within 36 h for remotely collected samples. Haematocrit was determined manually using standard capillary tube and centrifugation methods. The WBC was assessed using a haemocytometer and Phloxine B solution. Total leucocyte count was performed manually by use of a direct leucocyte counting method. Briefly, a Phloxine stain formulation was prepared using 0.5 g Phloxine stain powder (Phloxine B powdered stain-25 g; Sigma-Aldrich, St Louis, MO, USA) and 250 ml of 1,2-propanediol, 99.5% (Sigma-Aldrich), brought up to 500 ml with deionized H_2_O. Phloxine stain solution (620 µl) and 20 µl of the blood sample (1:32 dilution) were placed into a labelled, dated test tube. The test tube was placed on a tube rocker for 10 min at room temperature. This mixture was then loaded onto a Neubauer haemacytometer and the stained leucocytes were counted. The indirect total white blood cell count was calculated as follows: number of cells counted multiplied by the depth and the dilution, divided by the number of squares counted to produce the ‘A’ count (heterophil/eosinophil count). The ‘A’ count was then divided by the percentage of heterophils plus the percentage of eosinophils obtained from the manual differential and multiplied by 100. Differential white blood cell counts were performed by a board-certified veterinary clinical pathologist (D.D.). One hundred white blood cells were identified on fixed blood smears stained with an automated stainer (HemaTek, 2000; Bayer Health Care, Tarrytown, NY, USA) using Modified Wright’s Giemsa stain (Fisher Scientific, Middletown, VA, USA). Absolute heterophil, eosinophil, lymphocyte and monocyte counts were calculated by multiplying the relative percentage of each cell type by the total WBC. Of this set of complete blood count data, only the WBC (total WBC count; in cells per millilitre) and the heterophil-to-lymphocyte ratio (H/L ratio; based on cells per millilitre) were analysed statistically, because these two measures are known to be affected rapidly by corticosterone (Davis *et al.*, 2008). However, additional complete blood count data are also described here because there are limited published haematological data for this species.

#### Corticosterone assay

Unextracted plasma samples were assayed for corticosterone using a double-antibody ^125^I radioimmunoassay kit that has previously been validated for unextracted plasma from this species (Hunt *et al.*, 2012; catalogue number 07-120103; MP Biomedicals, Solon, OH, USA). Samples were diluted 10-fold in assay buffer before analysis in order to bring results closer to 50% bound on the standard curve, the area of greatest assay precision, with final results then multiplied by 10. The manufacturer’s reported cross-reactivity for this assay is 1% for testosterone and <1% for all other tested steroids. Intra-assay variation in our laboratory for this species is 4.6%; inter-assay variation is 9.7%, and assay sensitivity is 20 pg/ml. The manufacturer’s protocol was used except that all samples, standards and reagents were used at half-volume. Non-specific binding tubes and blanks were assayed in quadruplicate, and standards, controls and samples in duplicate. Any samples with a coefficient of variation >10% between duplicate tubes or that fell outside 10–90% bound were rediluted and reassayed accordingly. For further assay details, see Hunt *et al.* (2012).

### Data analysis

Variables were divided into the following three groups for analysis: nine clinical health measures (pH, pO_2_, pCO_2_, bicarbonate, sodium, potassium, ionized calcium, lactate and haematocrit), four stress-associated variables (corticosterone, glucose, WBC and H/L ratio) and vital signs (heart rate, respiratory rate and cloacal temperature). Lactate results were sometimes below the detectability limit of 0.30 mmol/l; such samples were assigned a nominal value of 0.15 mmol/l lactate, half of the detectability limit. Lactate, haematocrit and corticosterone data were logarithmically transformed before analysis to adjust for non-normal distributions based on skewness and kurtosis. Three of the 13 h turtles had missing iCa data for one of their four samples, i.e. the i-STAT machine reported no result. All data were inspected by a veterinarian (C.I.) for any clinically relevant deviations from expected values for healthy individuals compared with data reported by Innis *et al.* (2007, 2009) for juveniles of this species.

Descriptive statistics (means ± SEM) were used to summarize the data set. To test whether adrenal stress responses occurred during transportation, we performed repeated-measures multivariate analysis of variance (ANOVA) on the four stress-associated measures of corticosterone, glucose, WBC and H/L ratio. The model compared the repeated-measures variables of treatment (control and transport days), time (pre- and post-treatment events) and their interaction. The nine clinical health measures were investigated using repeated-measures ANOVA including independent factors of treatment and time, but using univariate analyses rather than a multivariate analysis because of missing data for calcium for some turtles (see paragraph above). Data sets for the 13 and 26 h groups were analysed separately. A significant interaction of treatment × time was termed a ‘transport effect’ and a significant effect of time was termed a ‘handling effect’. The assumption of sphericity was met in all analyses. The value of α (significance threshold) was set initially at 0.05. For univariate analyses, α was then adjusted using the false-discovery-rate method to correct for multiple univariate comparisons, e.g*.* all univariate analyses from the same set of animals were first ranked by *P*-value (lowest to highest), and then each *P*-value was compared with an individualized significance threshold, *d*, where *d* = 0.05 × rank/number of analyses (Curran-Everett, 2000, 2004). Data were analysed using the statistical software SPSS (v20.0.0 for Macintosh OSX) and Prism (6.0g for Macintosh OSX).

## Results

### Clinical health measures and vital signs

After multiple-comparisons correction, no significant transport effect or handling effect was seen for any of the nine clinical health measures (pH, pO_2_, pCO_2_, bicarbonate, sodium, potassium, ionized calcium, lactate or haematocrit) for either transport duration (Tables 1 and 2). Vital signs and other haematological data remained within expected limits for healthy juveniles of this species (Tables 3 and 4).

**Table 1:** Data from the turtles transported for 13 h

Parameter	Pre-control	Post-control	Pre-transport	Post-transport	Transport	Handling
					*P*-value	*P*-value
Clinical health parameters						
pH	7.54 ± 0.01	7.51 ± 0.01	7.49 ± 0.01	7.49 ± 0.01	0.2076	0.3837
pO_2_ (mm Hg)	67.9 ± 2.19	62.3 ± 2.03	62.8 ± 2.18	69.5 ± 2.85	0.0060	0.7829
pCO_2_ (mm Hg)	39.8 ± 0.79	41.0 ± 1.16	44.1 ± 1.45	41.3 ± 1.69	0.0988	0.5776
HCO_3_ (mmol/l)	38.8 ± 1.03	37.8 ± 1.02	37.5 ± 1.01	36.5 ± 0.81	0.8481	0.0618
Na (mmol/l)	148.9 ± 0.79	150.1 ± 0.57	150.1 ± 0.58	149.2 ± 0.68	0.0392	0.8279
K (mmol/l)	3.4 ± 0.04	3.8 ± 0.13	3.9 ± 0.11	3.6 ± 0.16	0.0141	0.5086
iCa (mmol/l)	0.9 ± 0.02	0.9 ± 0.03	0.9 ± 0.03	0.9 ± 0.02	0.2125	0.0584
Lactate (mmol/l)	0.35 ± 0.12	1.29 ± 0.38	1.09 ± 0.38	0.95 ± 0.44	0.0663	0.2176
Haematocrit (%)	29.3 ± 0.56	28.5 ± 0.60	29.4 ± 0.90	30.7 ± 0.72	0.0122	0.4935
Stress-associated parameters						
Glucose (mg/dl)	110 ± 2.0	115 ± 3.2	100 ± 2.8	137 ± 8.5	0.0020*	<0.0001*
WBC	4.4 ± 0.23	5.3 ± 0.65	5.7 ± 0.49	6.5 ± 1.14	0.9250	0.1950
H/L ratio	2.1 ± 0.18	2.7 ± 0.33	2.2 ± 0.40	4.9 ± 1.5	0.2380	0.0650
Corticosterone (ng/ml)	2.85 ± 0.44	3.18 ± 0.34	5.10 ± 1.42	7.05 ± 2.82	0.8650	0.2450

Means (±SEM) for eight clinical health variables and four stress-associated variables, before and after a 13 h control handling event (pre-control and post-control) and before and after a 13 h transport event (pre-transport and post-transport) of 15 Kemp’s ridley sea turtles. The same turtles were used on control days and on transport days. Definitions: ‘transport *P*-value’, *P*-value for the interaction of treatment × time; and ‘handling *P*-value’, *P*-value for a main effect of time. *P*-values are derived from two-factor ANOVAs; only those *P*-values indicated with an asterisk were significant after correction for multiple comparisons. Abbreviations: iCa, ionized calcium; WBC, total white blood cell count; H/L ratio, heterophil/lymphocyte ratio.

**Table 2:** Data from the turtles transported for ∼26 h

Parameter	Pre-control	Post-control	Pre-transport	Post-transport	Transport	Handling
					*P*-value	*P*-value
Clinical parameters						
pH	7.528 ± 0.03	7.515 ± 0.03	7.534 ± 0.01	7.551 ± 0.02	0.4400	0.9399
pO_2_ (mm Hg)	66.3 ± 2.5	71.8 ± 4.0	70.4 ± 2.8	64.5 ± 3.2	0.0968	0.9525
pCO_2_ (mm Hg)	38.6 ± 2.4	37.7 ± 2.5	37.5 ± 0.6	33.1 ± 1.3	0.2880	0.2460
HCO_3_ (mmol/l)	37.4 ± 1.2	35.5 ± 1.0	37.2 ± 0.9	35.3 ± 1.2	0.9670	0.0101
Na (mmol/l)	152.4 ± 0.6	152.9 ± 0.4	151.0 ± 0.9	152.2 ± 0.9	0.5984	0.2138
K (mmol/l)	3.8 ± 0.2	3.8 ± 0.2	3.5 ± 0.1	3.6 ± 0.3	0.7590	0.5241
iCa (mmol/l)	0.86 ± 0.02	0.83 ± 0.02	0.85 ± 0.02	0.80 ± 0.03	0.3659	0.0366
Lactate (mmol/l)	1.56 ± 0.87	1.09 ± 0.46	0.34 ± 0.13	1.79 ± 0.98	0.2351	0.4259
Haematocrit (%)	28.0 ± 0.53	27.2 ± 0.97	26.6 ± 0.81	35.6 ± 2.17	0.0027	0.0012
Stress-associated parameters						
Glucose (mg/dl)	104 ± 2.8	111 ± 2.8	107 ± 1.9	143 ± 11.4	0.0400*	0.0030*
WBC	6.1 ± 0.5	7.6 ± 0.9	7.8 ± 0.7	10.9 ± 1.9	0.4290	0.0340*
H/L ratio	1.08 ± 0.18	1.11 ± 0.13	1.41 ± 0.2	2.80 ± 0.7	0.1190	0.1150
Corticosterone (ng/ml)	1.82 ± 0.20	3.22 ± 0.41	2.22 ± 0.31	11.56 ± 3.42	0.0020*	<0.0001*

Means (±SEM) for eight clinical health variables and four stress-associated variables, before and after a 23 h control handling event (pre-control and post-control) and before and after a 26 h transport event (pre-transport and post-transport) of 11 Kemp’s ridley sea turtles. The same turtles were used on control days and on transport days. Definitions: ‘transport *P*-value’, *P*-value for the interaction of treatment × time; and ‘handling *P*-value’, *P*-value for a main effect of time. *P*-values are derived from two-factor ANOVAs; only those *P*-values indicated with an asterisk were significant after correction for multiple comparisons. Abbreviations: iCa, ionized calcium; WBC, total white blood cell count; H/L ratio, heterophil/lymphocyte ratio.

**Table 3:** Vital signs, timing parameters and haematological data for the turtles transported for 13 h

Parameter	Pre-control	Post-control	Pre-transport	Post-transport
Vital signs				
Temperature (°C)	26.86 ± 0.06	26.35 ± 0.07	27.77 ± 0.06	25.63 ± 0.33
Heart rate (beats/min)	52.80 ± 1.25	51.60 ± 1.05	55.00 ± 1.74	48.00 ± 1.35
Respirations (breaths/min)	4.85 ± 0.59	3.93 ± 0.34	3.03 ± 0.38	4.40 ± 0.41
Timing parameters (min; measured from time of first disturbance)				
Bleeding time	1.94 ± 0.08	3.05 ± 0.35	2.33 ± 0.36	1.36 ± 0.18
Handling time	11.98 ± 0.72	9.63 ± 0.53	Not recorded	Not recorded
CG4 lag time	2.54 ± 0.50	1.52 ± 0.14	2.19 ± 0.26	2.52 ± 0.39
CG8 lag time	7.01 ± 0.81	5.24 ± 0.14	5.69 ± 0.22	6.07 ± 0.39
Haematological data				
Heterophils (%)	64.87 ± 1.97	68.93 ± 2.49	63.87 ± 2.69	71.80 ± 3.69
Lymphocytes (%)	32.47 ± 1.73	29.00 ± 2.41	34.60 ± 2.68	26.27 ± 3.52
Monocytes (%)	1.93 ± 0.32	1.33 ± 0.21	1.27 ± 0.25	1.53 ± 0.27
Eosinophils (%)	0.73 ± 0.18	0.67 ± 0.23	0.27 ± 0.12	0.40 ± 0.21
Heterophils (cells/µl)	2877 ± 202.6	3789 ± 570.4	3784 ± 458.0	5045 ± 1109.9
Lymphocytes (cells/µl)	1412 ± 87.9	1466 ± 152.2	1873 ± 141.8	1386 ± 125.9
Monocytes (cells/µl)	85 ± 15.8	63 ± 10.6	75 ± 18.0	92 ± 17.2
Eosinophils (cells/µl)	33 ± 8.4	26 ± 8.2	14 ± 6.5	17 ± 9.8

Data are presented as means ± SEM.

**Table 4:** Vital signs, timing parameters and haematological data for the turtles transported for 26 h

Parameter	Pre-control	Post-control	Pre-transport	Post-transport
Vital signs				
Temperature (°C)	24.52 ± 0.14	24.44 ± 0.10	24.83 ± 0.07	22.52 ± 0.20
Heart rate (beats/min)	48.83 ± 0.94	46.00 ± 0.95	48.00 ± 1.21	37.50 ± 1.73
Respirations (breaths/min)	4.83 ± 0.71	3.79 ± 0.47	3.75 ± 0.42	3.29 ± 0.40
Timing parameters (min; measured from time of first disturbance)				
Bleeding time	2.66 ± 0.55	2.42 ± 0.35	1.69 ± 0.06	1.22 ± 0.18
Handling time	7.63 ± 0.57	6.83 ± 0.60	Not recorded	Not recorded
CG4 lag time	2.33 ± 0.38	1.59 ± 0.15	2.22 ± 0.58	1.81 ± 0.20
CG8 lag time	6.45 ± 0.42	5.44 ± 0.17	5.53 ± 0.16	5.57 ± 0.21
Haematological data				
Heterophils (%)	48.0 ± 4.01	50.0 ± 2.99	54.0 ± 2.97	66.1 ± 3.11
Lymphocytes (%)	47.8 ± 4.04	46.7 ± 2.75	43.1 ± 2.91	31.5 ± 2.97
Monocytes (%)	2.0 ± 0.33	1.7 ± 0.47	2.4 ± 0.56	1.8 ± 0.46
Eosinophils (%)	2.1 ± 0.54	1.7 ± 0.51	0.5 ± 0.23	0.1 ± 0.08
Heterophils (cells/µl)	2686 ± 231.3	3821 ± 530.6	4170 ± 479.1	7445 ± 1722.5
Lymphocytes (cells/µl)	2966 ± 439.4	3463 ± 359.2	3204 ± 294.2	2861 ± 196.2
Monocytes (cells/µl)	104 ± 13.0	155 ± 67.7	172 ± 42.4	159 ± 37.9
Eosinophils (cells/µl)	122 ± 38.1	111 ± 37.0	37 ± 18.4	5 ± 5.3

Data are presented as means ± SEM.

### Stress-associated measures

Turtles transported for 13 and 26 h were found to have a significant physiological stress response (multivariate ANOVA of all four stress-related measures; 13 h transport, *F*_4,11_ = 10.367, *P* = 0.001; 26 h transport, *F*_4,7_ = 5.797, *P* = 0.022).

Of all variables studied, glucose showed the most consistent effects of both transport and handling. Glucose showed a clear and significant effect of transport (mean elevation of 36% after the 13 h transport and 33% after the 26 h transport; Tables 1 and 2). Glucose also showed a significant handling effect, with a small but consistent increase from the pre to the post sample even on control days, elevating by a mean of 4% during the 13 h control day and 7% during the 26 h control day (Tables 1 and 2).

Corticosterone showed strong and significant effects of both transport and handling in the 26 h turtles, with similar (though non-significant) trends in the 13 h turtles. In the 26 h turtles, corticosterone nearly doubled on the control day, increasing in 10 of 11 turtles, and increased more than 5-fold on transport day, increasing in all 11 turtles (Tables 1 and 2). The 13 h turtles had a weak elevation of 11% in mean corticosterone on the control day and a more pronounced increase of 38% on the transport day; these changes were not always consistent on a within-turtle basis, however, with 10 of the 15 13 h turtles showing an increase in corticosterone on the control day and only eight of 15 on the transport day.

Both WBC and H/L ratio tended to elevate during the transport days and (to a lesser degree) during the control days, but these trends were erratic and usually non-significant (Tables 1 and 2). WBC was significantly affected by handling in the 26 h turtles but not in the 13 h turtles, and transport had no significant effect on WBC. The H/L ratio showed no statistically significant effects of transport or handling.

## Discussion

Generally, juvenile Kemp’s ridley sea turtles showed a mild stress response to transportation of 13 h and a more pronounced stress response to an overnight transport lasting 26 h. However, clinical health measures and vital signs remained within expected limits for both durations of transport and for all turtles.

### Baseline corticosterone

Baseline corticosterone of pre-control samples was generally 2–3 ng/ml in this study. Baseline corticosterone for Kemp’s ridley sea turtles has variously been reported as ∼0.10 (Ortiz *et al.*, 2000), ∼2 (Morris, 1982), between 0.2 and 4.0 (Stephenson *et al.*, 1998) and ∼6 ng/ml (Gregory and Schmid, 2001); in the last of these studies, exclusion of two outliers brought mean corticosterone to ∼3 ng/ml. These studies span a wide range of corticosterone concentrations, possibly as a result of variation in assay methodology or handling protocols, but generally, our results agree well with those of Morris (1982) and Gregory and Schmid (2001). Mean baseline corticosterone of other sea turtle species also spans a wide range between ∼0.1 and 4.0 ng/ml, often with significant effects of sex, season, age and reproductive state (flatback, *Natator depressus*, Ikonomopoulou *et al.*, 2014; green turtle, *Chelonia mydas*, Jessop *et al.*, 1999, 2000, 2004a,b; Rostal *et al.*, 2001; Jessop and Hamann, 2004; hawksbill, *Eretmochelys imbricata*, Jessop *et al.*, 2004c; Jessop and Hamann, 2005; leatherback, *Dermochelys coriacea*, Deem *et al.*, 2006; loggerhead, *Caretta caretta*, Gregory *et al.*, 1996; Whittier *et al.*, 1997; olive ridley, *Lepidochelys olivacea*, Valverde, 1996; Valverde *et al.*, 1999; Blanvillain *et al.*, 2008; Valente *et al.*, 2011). It should be noted that some turtles in our study may have had altered adrenal activity owing to such factors as anticipatory stress (e.g. turtles may have detected alterations in morning routine, such as more personnel, increased human activity and nets being carried nearby) or generally increased stress because of the captive environment. However, given that all individuals were in good health, exhibited apparently normal behaviour (including vigorous swimming and eating) and had many months of acclimation to the captive environment, we consider that the pre-control corticosterone concentrations reported here probably represent close-to-baseline corticosterone for juvenile Kemp’s ridley sea turtles. Additionally, the average bleeding time (∼2 min) in the present study was short and is likely to have minimized artifactual changes due to handling (Romero and Reed, 2005).

### Transport elicits a stress response

In the 13 h transport, corticosterone was slightly elevated in the post-transport samples compared with the pre-transport samples, but after multiple-comparisons correction this elevation was not significant. However, glucose was significantly elevated after the 13 h transport. Given that glucose is strongly affected by adrenal hormone concentrations of the previous hours (Widmaier, 1990; Mizock, 1995) and is known to elevate in response to stress in juvenile Kemp’s ridley sea turtles specifically (Gregory and Schmid, 2001), we surmise that the 13 h turtles may have experienced a brief, acute elevation in corticosterone during transport that had returned to near-baseline by the time the transport ended. Overall, transport of 13 h appears to be, at most, a mild stressor, given the transport protocols observed in the present study (controlled temperature and padded individual crates; see below).

The 26 h transport event elicited a much stronger stress response. Corticosterone was significantly and strongly elevated, to a mean of 11.6 ng/ml, more than five times baseline. It should be noted that this corticosterone concentration is lower, on an absolute basis, than that seen during extremely stressful events, such as cold-stunning and stranding (Hunt *et al.*, 2012), although reported baselines in various studies differ. For example, wild Kemp’s ridley sea turtles that were captured in nets and then placed on their backs (‘turning stress’) for 60 min had an average plasma corticosterone of 24.68 ± 3.65 ng/ml (Gregory and Schmid, 2001), which in that instance represented a 5-fold elevation above the baseline measured in that study. Cold-stunned Kemp’s ridley sea turtles admitted to our clinic in previous years have been found to have plasma corticosterone of 39.3 ± 2.5 ng/ml on the day of admission, with some individuals >80 ng/ml (e.g. after a combination of stressors that include cold-stunning, stranding, handling and then road transport to the clinic), approximately 20–30 times higher than concentrations commonly seen after a month of rehabilitation (Hunt *et al.*, 2012). Finally, Kemp’s ridley sea turtles exposed to an osmotic stressor (exposure to freshwater for 2 days) experienced a 3.3-fold increase in corticosterone over baseline (Ortiz *et al.*, 2000). A variety of stressors have been studied in other sea turtle species as well. Olive ridley sea turtles (*Lepidochelys olivacea*) have been reported to show an approximately 15-fold elevation in corticosterone after 6 h of turning stress (Valverde *et al.*, 1999). Green turtles show an approximately 4-fold increase in corticosterone after capture and placement in a saltwater tank (Aguirre *et al.*, 1995) or a 16-fold increase if heating stress is added to capture stress (Jessop *et al.*, 2000). Loggerheads exhibit a 7-fold increase in corticosterone in response to capture and holding out of water for up to 20 h (Blanvillain *et al.*, 2008) and a 10-fold increase for capture and overheating (Gregory *et al.*, 1996). Hawksbills have been reported to have an 11- to 19-fold increase (depending on sex) in corticosterone after 5 h of turning stress (Jessop *et al.*, 2004b). Comparisons across species, sexes, ages and different methodologies are difficult, but in general the 5- to 6-fold increase in corticosterone measured in our study appears to be a moderate but not a maximal stress response.

Glucose showed a consistent elevation after transport in the present study, generally increasing to the 130–140 mg/dl range. Similar patterns of increased glucose are seen in livestock in response to road transport and other types of stressors and are generally interpreted as evidence of a physiological stress response (Exton, 1979; McMahon *et al.*, 1988; Knowles and Warriss, 2007). Glucose concentrations have not been widely reported in sea turtle stress studies, but Gregory and Schmid (2001) reported initial plasma glucose concentrations of 94.41 ± 3.78 mg/dl in Kemp’s ridley sea turtles captured in entanglement nets, elevating to 106.43 ± 4.82 mg/dl after 60 min of turning stress. Two other studies report glucose >100 mg/dl in Kemp’s ridley sea turtles, in both cases after exposure to a known stressor; Hoopes *et al.* (2000) described glucose concentrations between 113 ± 5.4 and 129.6 ± 9.0 mg/dl (means ± SEM) for two groups of Kemp’s ridley sea turtles that had been captured in entanglement nets for up to 45 min before sampling, whereas Snoddy *et al.* (2009) reported glucose concentrations of 112.3 ± 24.4 mg/dl for four Kemp’s ridley sea turtles that had been in entanglement nets for up to 2 h. Finally, green turtles subjected to known stressors had glucose concentrations comparable to those reported here, with ∼140 mg/dl mean glucose after 4 h of turning stress (Aguirre *et al.*, 1995) and ∼135–140 mg/dl mean glucose after entanglement of up to 2 h (Snoddy *et al.*, 2009).

Taking the corticosterone and glucose data together, transport of 13 h appears to be only a minor stressor (given the handling and transport protocols observed here; see below). We suggest that the changes in corticosterone and glucose seen in the 26 h transport can probably be considered indications of an intermediate but not a maximal stress response.

### Turtles remain in good clinical health even after 26 h transport

There are no well-established reference intervals for Kemp’s ridley sea turtle blood data; however, a number of studies provide comparative data for presumed healthy wild or captive rehabilitated Kemp’s ridley sea turtles (Stabenau *et al.*, 1991; Carminati *et al.*, 1994; Moon *et al.*, 1997; Turnbull *et al.*, 2000; Innis *et al.*, 2007, 2009; Kimmel, 2007; Keller *et al.*, 2012). All health data in the present study were consistent with these previous studies, and despite the moderate stress of even the longer transport, all health measures remained within clinically acceptable limits. For example, the increases in glucose concentrations during transport were minimal in comparison to the derangements that are often seen in debilitated, hospitalized Kemp’s ridley turtles (Innis *et al.*, 2009; Keller *et al.*, 2012).

Transport days included subcutaneous fluid therapy, whereas control days did not. This difference in protocol was due to the fact that turtles were out of water during the entire transport event, whereas on control days the turtles remained in water. Despite this difference, the hydration status of the turtles was similar and clinically normal on control and transport days based on evaluation of sodium and haematocrit data. Additional analytes, including plasma total protein and uric acid concentrations, could have provided more information about the hydration status of the turtles. Likewise, other biochemical parameters, such as plasma enzyme activities, could have been used to assess for potential cellular injury resulting from transport.

### Handling stress and anticipation

In the case of the stress-associated variables, it seems likely that handling and food limitation represent mild stressors that may elicit a very mild stress response even on the control day, although this effect was dwarfed by the more pronounced changes seen on transport days (Tables 1 and 2). Interestingly, several stress-associated parameters also showed a mild increase in the pre-transport sample compared with the pre-control sample 2 weeks earlier (Tables 1 and 2), perhaps indicating anticipation or learning on the part of the turtles; that is, they may have learned to recognize certain early morning activities (e.g. early arrival of staff, increased activity near tanks as equipment is set up) as indications that a handling event is about to happen (as described by Harper and Austad 2000; Moreira and Volpato, 2004). Minor deviations in the time of day of the various events may also have influenced these patterns.

### Point-of-care methodology

The present study provides the first blood data for Kemp’s ridley sea turtles using the i-STAT point-of-care analyser. Although there is ongoing debate over the validity of this analyser for ectothermic animals (Harter *et al.*, 2015), the i-STAT appears to provide clinically relevant data for sea turtles based on many previous studies (Chittick *et al.*, 2002; Harms *et al.*, 2007, 2014; Innis *et al.*, 2010, 2014; Anderson *et al.*, 2011; Lewbart *et al.*, 2014), and it is clear that data generated by other point-of-care analysers are of relevance to clinical assessment and prognosis of stranded Kemp’s ridley sea turtles (Innis *et al.*, 2007; Keller *et al.*, 2012; Stacy *et al.*, 2013). Data in the present study were generally consistent with previously documented values for healthy Kemp’s ridley sea turtles, acknowledging the expected minor differences among various biochemical analysers (Wolf *et al.*, 2008; Atkins *et al.*, 2010). The lag time before initiation of blood analysis was short (generally within 2–6 min after collection), consistent with the manufacturer’s guidelines, and thus is likely to have minimized any artifactual changes.

### Implications for transport logistics

Although the stable clinical health of these turtles after even a 26 h transport is encouraging, it is also important to recognize that a moderate stress response might entail some detrimental effects for those turtles over the next days or weeks. Prolonged stress responses (i.e. more than a few hours) can have negative impacts on health, growth, survival and immune response that may have delayed effects (Wingfield *et al.*, 1997; Sapolsky *et al.*, 2000; Cabezas *et al.*, 2007). It is unknown at present to what degree a ‘moderate’ stress response, such as that seen in the 26 h transport, might affect a newly released sea turtle over the next several days to weeks, but it seems prudent to seek to minimize such stress where possible. Given the corticosterone and glucose patterns seen after 26 compared with 13 h of transport, we believe it advisable to minimize transport duration as much as possible. This can involve additional expense and staff, which may entail logistical complications, such as multiple teams of drivers (to rotate driving shifts through the night), additional vehicles to carry the extra staff, minimization of meal stops and streamlining of release logistics at the release beach. For example, it is not uncommon for beach releases to involve media and spectators that can greatly delay the release, even after turtles have already experienced a long transport; such events, although valuable from an outreach perspective, should be expedited where possible.

We emphasize that our results are likely to be dependent to some degree on the habituation of turtles to human contact during the months of rehabilitation, as well as particular transport and handling protocols used by clinical staff and the transport team. Additional studies are required to determine whether turtles that have not been in rehabilitation for extended periods (i.e. more recently stranded turtles) could be more severely affected by transport. In our transport events, all logistics are planned with the goal of streamlining the transport and minimizing stress on the animals. Features of our transport protocol that may be relevant include the following: lubrication of the turtle’s skin; subcutaneous fluid therapy on the morning of transport; individual crates with padding; air holes providing adequate ventilation; ambient temperature within the vehicle set to 26°C (monitored with a remote temperature probe where possible); turtles handled gently and briefly, and not moved excessively; total handling time typically <10 min per turtle; noise minimized in the vehicle; and every reasonable effort made to conclude the transport rapidly and efficiently. Assuming that such protocols are observed, it appears that juvenile Kemp’s ridley sea turtles should tolerate transports of up to 26 h relatively well.

In conclusion, transport events up to 26 h for juvenile Kemp’s ridley sea turtles seem to represent a moderate stressor, but one that does not adversely affect health in the short term. Further data are needed on shorter (<13 h) and longer (>26 h) transports, transports of more recently stranded turtles and on other species of sea turtle. For example, preliminary data indicate that other species of sea turtle (green and loggerhead) may react in a different manner to transportation (K. Hunt, C. Innis and C. Merigo, unpublished data). Body size and age may also be relevant; turtles in the present study were relatively small, and it is possible that turtles of greater body mass may experience more difficulty in breathing for extended periods out of water. Finally, it would be useful to explore methods of recovery after transport before releasing the turtles to the sea (e.g. putting turtles in a saltwater tank overnight at a site near release beaches). Until more data are available on the potential effects of longer transports, we tentatively suggest that vehicle transports longer than 26 h be used cautiously, and only when unavoidable. In general, we emphasize that although transport stress cannot be eliminated, it can and should be minimized where possible. The findings of the present study may be of use for future comparison with sea turtles transported during other rescue and rehabilitation efforts.

## Funding

This work was supported by Industrial Economics, Inc., under contract of the National Oceanographic and Atmospheric Administration (contract number AB133C-11-CQ-0050).
